# Three new species in *Cordycipitaceae* and *Clavicipitaceae* (*Hypocreales*, *Ascomycota*) from Yunnan, China

**DOI:** 10.3897/mycokeys.137.195207

**Published:** 2026-07-23

**Authors:** Zhi-Qin Wang, Zi-Han Wang, Yu-Xin Zhang, Qian Song, Wen-Ya Shao, Wen-Li Li, Yuan-Bing Wang

**Affiliations:** 1 College of Biological Science and Food Engineering, Southwest Forestry University, Kunming 650224, China State Key Laboratory of Phytochemistry and Natural Medicine, Kunming Institute of Botany, Chinese Academy of Sciences Kunming China; 2 State Key Laboratory of Phytochemistry and Natural Medicine, Kunming Institute of Botany, Chinese Academy of Sciences, Kunming 650201, China Yunnan Key Laboratory for Fungal Diversity and Green Development, Kunming Institute of Botany, Chinese Academy of Sciences Kunming China; 3 Yunnan Key Laboratory for Fungal Diversity and Green Development, Kunming Institute of Botany, Chinese Academy of Sciences, Kunming 650201, China College of Biological Science and Food Engineering, Southwest Forestry University Kunming China

**Keywords:** *
Ascopolyporus
*, *
Cordyceps
*, *
Hypocrella
*, morphology, new species, phylogenetic analyses

## Abstract

Yunnan Province is a hotspot for biodiversity research. In recent years, the diversity of entomopathogenic fungi in this region has been continuously reported. During a survey of entomopathogenic fungi in Yunnan Province, three new species, *Ascopolyporus sinensis*, *Cordyceps anningensis*, and *Hypocrella umbilicata*, were identified and proposed based on multigene phylogenetic analyses and morphological characterization. Phylogenetic analyses were conducted using a combined dataset of nrSSU, nrLSU, *tef-1α*, *rpb1*, and *rpb2*, which clarified the taxonomic status of these three new species. *Ascopolyporus sinensis* is positioned at the base of the genus *Ascopolyporus (Cordycipitaceae)* and is characterized by the production of a pale purplish-pink pigment that diffuses into PDA plates and fusiform to acerose conidia aggregated at the apices of phialides. *Cordyceps anningensis* is phylogenetically closely related to *C. sandindaengensis* and *C. locastraeare* but can be distinguished from these two species by the position of the synnemata on the host insect and the size of the conidia. *Hypocrella umbilicata* is similar to closely related species of *Hypocrella (Clavicipitaceae)* in having pulvinate stromata and filiform to long-fusiform ascospores that do not disarticulate but differs in having a distinctly depressed central region on the stromata. The discovery of these three taxa contributes to our understanding of fungal diversity and phylogeny in this region.

## Introduction

Yunnan is an important biodiversity treasure trove and ecological security barrier in southwestern China. It possesses more than half of the country’s ecosystem diversity and biodiversity and is also one of the 36 global biodiversity hotspots with the richest species diversity (Yang et al. 2004; Hu et al. 2018). It is an important hotspot for diversity studies, and numerous studies have discovered new species with local distributions (Wang et al. 2020, 2025; Dong et al. 2022). Entomopathogenic fungi have a broad host range and can colonize hosts from more than 10 invertebrate orders (Sung et al. 2007; Fan et al. 2024).

Several genera of entomopathogenic fungi have been reported to induce epizootics in scale insects or whiteflies, including members of *Cordycipitaceae*, such as *Ascopolyporus* Möller and *Hyperdermium* J.F. White et al., and members of *Clavicipitaceae*, such as *Aschersonia* Mont., *Conoideocrella* D. Johnson et al., *Dussiella* Pat., *Helicocollum* Luangsa-ard et al., *Hypocrella* Sacc., *Moelleriella* Bres., *Regiocrella* P. Chaverri & K.T. Hodge, *Orbiocrella* D. Johnson et al., and *Samuelsia* P. Chaverri & K.T. Hodge (Thanakitpipattana et al. 2022; Wang et al. 2025). Among these, *Ascopolyporus* is an epiphytic fungal genus comprising 10 species, with *A. polychrous* Möller as the type species (Thanakitpipattana et al. 2022; Yu et al. 2023). Stromata of *Ascopolyporus* form on the stems of living plants and acquire nutrients through two distinct strategies: acting as biotrophs that utilize living plant stems and as necrotrophs that infect scale insects (Thanakitpipattana et al. 2022). Stromata of *Ascopolyporus* species exhibit considerable morphological diversity, particularly in shape and color. The genus is characterized by semi-immersed to immersed perithecia, filiform ascospores, and aseptate to septate conidia that aggregate at the apices of phialides.

Another genus, *Hypocrella* sensu lato (s. l.) (anamorph *Aschersonia* s. l.), was erected by Saccardo in 1878 to accommodate four species. Chaverri et al. (2008) revised *Hypocrella* s. l. species based on three-gene phylogenetic analyses combined with morphological characters, classifying them into *Hypocrella* sensu stricto (s. s.), *Moelleriella*, and *Samuelsia*. *Hypocrella* s. s. is characterized by filiform to long-fusiform ascospores that do not disarticulate, and *Aschersonia* s. s. anamorphs have fusoid conidia (Chaverri et al. 2008).

Based on multigene phylogenetic classification, Sung et al. (2007) revised *Cordyceps* s. l. and *Clavicipitaceae* s. l., proposing four genera: *Cordyceps* s. s. within *Cordycipitaceae*, *Metacordyceps* G.H. Sung et al. within *Clavicipitaceae*, and *Ophiocordyceps* Petch and *Elaphocordyceps* G.H. Sung & Spatafora within *Ophiocordycipitaceae*. The study ultimately accepted 45 species and established new combinations within *Cordyceps* s. s. (Sung et al. 2007). *Cordyceps* s. s. is a very important fungal resource because some species in this genus have edible and medicinal value, including *C. militaris* Fr., *C. cicadae* (Miq.) Massee, and *C. tenuipes* (Peck) Kepler et al. (Dong et al. 2022). Currently, the genus includes more than 600 recorded species (Index Fungorum: https://www.indexfungorum.org, 17 March 2026). Morphologically, *Cordyceps* s. s. species produce pallid or brightly pigmented stromata or subicula, superficial to completely immersed perithecia, cylindrical asci with thickened ascus apices, and cylindrical, multiseptate ascospores that may or may not disarticulate into part-spores (Sung et al. 2007).

During an investigation of the diversity of entomopathogenic fungi in Yunnan Province, China, several specimens were collected, and fungal strains were isolated and purified. These specimens and strains were identified based on multigene molecular phylogenetic analyses and morphological characteristics. As a result, three new species are introduced, namely, *Ascopolyporus sinensis*, *Cordyceps anningensis*, and *Hypocrella umbilicata*. Morphological descriptions and detailed comparisons with allied taxa are provided for the three new species in this study.

## Materials and methods

### Fungal collection and isolation

Samples of entomopathogenic fungi were collected in 2025 from Centipede Mountain, Anning City, and Lianhuatang Village, Simaogang Town, Pu’er City, Yunnan Province, China. The collected specimens were placed in sterilized plastic tubes or storage bags, stored in a refrigerator at 4 °C, and brought to the laboratory for further experiments. Fungal isolation from sexual specimens was performed following the detailed procedure described by Wang et al. (2020). The stromata were immersed in 30% H2O2 for 30 s and then rinsed five times with sterilized water. After drying on sterilized filter paper, the segments were inoculated onto potato dextrose agar (PDA) plates. For asexual specimens, conidia sporulating from synnemata were streaked onto PDA plates containing 0.1 g/L chloramphenicol. Subsequently, the cultures were purified. The plates were examined for contamination, and the cultures were transferred to new PDA plates. Specimens and pure cultures were deposited in the Cryptogamic Herbarium of the Kunming Institute of Botany (**KUN-HKAS**) and the Kunming Institute of Botany Culture Collection (**KUNCC**), Chinese Academy of Sciences, respectively.

### Morphological observations

Macromorphological characteristics and specimen photographs were examined and captured using an Olympus SZ60 stereomicroscope. Stroma size, texture, shape, and color were measured and recorded. Pure cultures were transferred to fresh PDA plates. Colony morphology in terms of color, shape, appearance, and growth was measured after incubation at 25 °C for 14 days. Sections of sexual stromata were prepared using a blade and mounted on slides with water or lactic acid–cotton blue for micromorphological observations. For observations of asexual morphs, slide cultures were prepared following the method described by Wang et al. (2020). This was performed by placing a small amount of mycelium on 3-mm-diameter PDA medium blocks overlaid with a coverslip. Micromorphological observations and measurements were conducted using an Olympus BX53 microscope.

### DNA extraction, PCR, and sequencing

Fungal mycelial mass was obtained from cultures grown on PDA. DNA extraction from fresh mycelia and dried stromatal tissue was performed using the Trelief Hi-Pure Plant Genomic DNA Kit TSP102-200 (Tsingke Biotechnology Co., Ltd., Beijing, China) following the manufacturer’s protocol. The extracted DNA was stored at −20 °C. The reaction mixture was prepared in a total volume of 25 µL and contained 12.5 µL of 2× Taq PCR Master Mix (Tiangen Biotech Co., Ltd., Beijing, China), 8.5 µL of sterile water, 1 µL each of the forward and reverse primers, and 2 µL of DNA template. The polymerase chain reaction (PCR) amplification procedure and primer pairs used for the nuclear ribosomal small and large subunits (nrSSU and nrLSU), translation elongation factor 1α (*tef-1α*), and the largest and second-largest subunits of RNA polymerase II (*rpb1* and *rpb2*) followed the description of Wang et al. (2020) (Suppl. material 2: tables S2, S3). Amplification reactions were performed using a Bio-Rad T100™ thermal cycler (Bio-Rad Laboratories, Hercules, CA, United States). The PCR products were purified and sequenced at Tsingke Biotechnology Co., Ltd. (Kunming, China).

### Sequence alignment and phylogenetic analyses

Newly generated sequences in this study were checked and edited using MEGA v. 6.06 before being deposited in the GenBank database (Tamura et al. 2013). Sequences used in this study were selected based on BLAST results from NCBI and previous studies (Dong et al. 2022; Thanakitpipattana et al. 2022; Crous et al. 2025) and were downloaded from GenBank (Suppl. material 2: table S1). Sequence alignments and the removal of poorly aligned regions were performed using MEGA v. 6.06 (Tamura et al. 2013). Single-gene alignment matrices for each gene were concatenated using PhyloSuite v. 1.2.2 (Zhang et al. 2020). All five loci were integrated into a unified dataset, which was further partitioned into 11 distinct segments for analysis. The optimal partitioning schemes and evolutionary models for each partition were determined using PartitionFinder2 (Lanfear et al. 2016), employing a greedy algorithm and selecting models based on the Akaike information criterion. The final 11 partitions and their corresponding optimal models were as follows: partition 1, nrSSU: TRN+I+G; partition 2, nrLSU: TRN+I+G; partition 3, *tef-1α* codon 1: TRN+I+G; partition 4, *tef-1α* codon 2: GTR+I+G; partition 5, *tef-1α* codon 3: K80+I+G; partition 6, *rpb1* codon 1: TVM+G; partition 7, *rpb1* codon 2: TVM+I+G; partition 8, *rpb1* codon 3: TIM+G; partition 9, *rpb2* codon 1: K81UF+I+G; partition 10, *rpb2* codon 2: K81UF+I+G; and partition 11, *rpb2* codon 3: GTR+G. Phylogenetic analyses were conducted using Bayesian inference (BI) and maximum likelihood (ML) methods implemented in MrBayes v. 3.2.2 and IQ-TREE v. 2.1.3, respectively (Ronquist and Huelsenbeck 2003; Stamatakis et al. 2008; Ronquist et al. 2012; Nguyen et al. 2015). The ML phylogenetic tree was constructed in a single run with 5,000 ultrafast bootstrap replicates (Hoang et al. 2017). The BI phylogenetic tree was constructed using four Markov chain Monte Carlo chains run for 2 million generations from a random starting tree, with a sampling frequency of 100 generations; the first 25% of samples were discarded as burn-in. Phylogenetic trees were edited and visualized using FigTree v. 1.4.3 and Adobe Illustrator CS6, respectively. The phylogenetic tree comprising a comprehensive sampling of *Cordyceps* species was constructed using the same method described above and is shown in Suppl. material 1: fig. S1.

## Results

### Sequencing and phylogenetic analyses

A total of 141 taxa from three families (*Clavicipitaceae*, *Cordycipitaceae*, and *Polycephalomycetaceae*), comprising six newly generated sequences and 135 sequences downloaded from GenBank, were used to conduct the phylogenetic analyses. *Pleurocordyceps aurantiaca* (Y.P. Xiao, T.C. Wen & K.D. Hyde) Y. Hui Wang et al. MFLUCC 17-2113 and *P. marginaliradians* (Y.P. Xiao, T.C. Wen & K.D. Hyde) Y. Hui Wang et al. MFLU 17-1582 from *Polycephalomycetaceae* were designated as outgroups. The combined five-gene dataset of 141 taxa consisted of 5,028 bp (nrSSU: 1,100 bp; nrLSU: 956 bp; *tef-1α*: 1,011 bp; *rpb1*: 809 bp; and *rpb2*: 1,152 bp).

**Figure 1.**
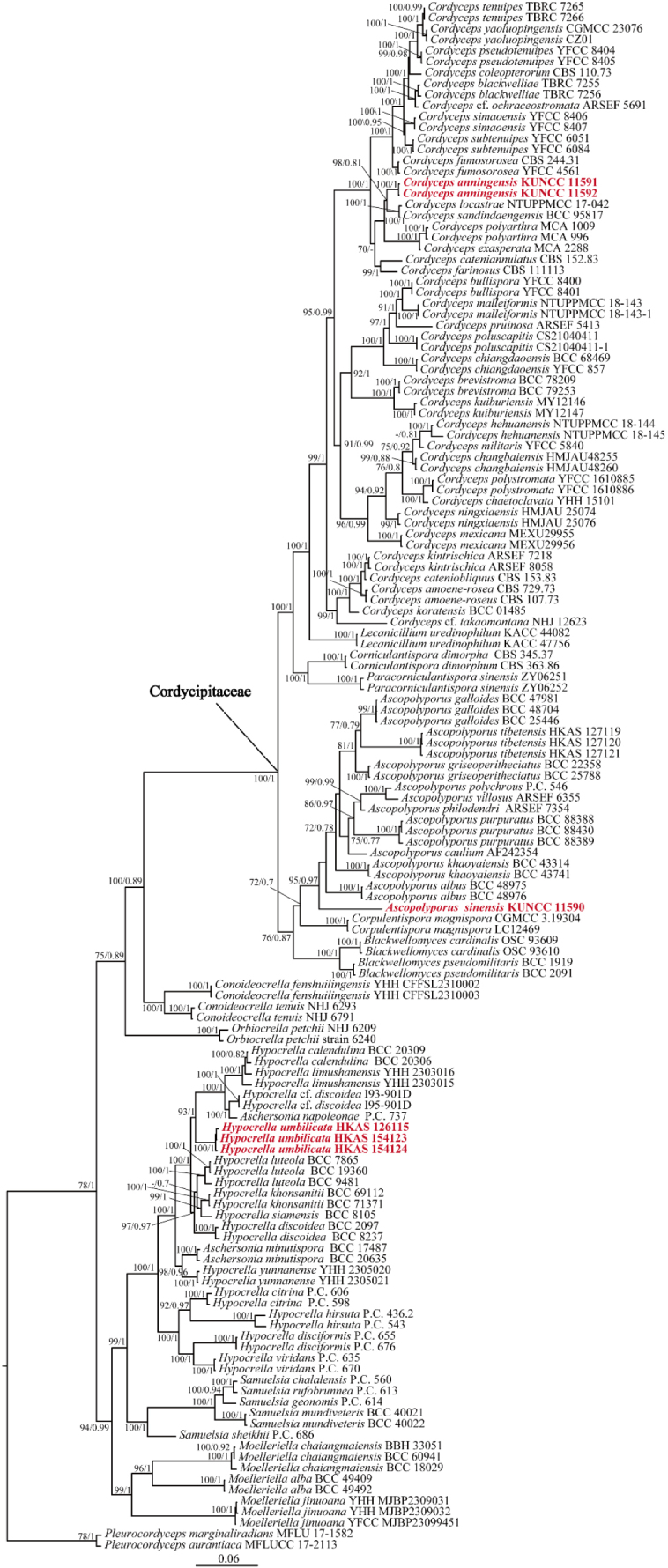
Phylogenetic tree of 12 genera within *Cordycipitaceae* and *Clavicipitaceae* based on the combined nrSSU, nrLSU, *tef-1α*, *rpb1*, and *rpb2* sequence dataset inferred using the ML and BI methods. Statistical support values greater than 70% are provided at the nodes as ML bootstrap proportions/BI posterior probabilities. New taxa are indicated in bold red font.

Phylogenetic trees were constructed using ML and BI methods to determine the phylogenetic placement of the new species. The two methods yielded consistent topologies with strong support (Fig. 1). The phylogenetic analysis revealed that the six newly generated sequences clustered within *Ascopolyporus* (*A. sinensis*KUNCC 11590), *Cordyceps* (*C. anningensis*KUNCC 11591 and KUNCC 11592), and *Hypocrella* (*H. umbilicata* HKAS 126115, HKAS 154123, and HKAS 154124). *Ascopolyporus sinensis*KUNCC 11590 formed an independent clade (BP = 95%, PP = 0.97) and was positioned at the base of the *Ascopolyporus* clade. *Cordyceps anningensis*KUNCC 11591 and KUNCC 11592 clustered into an independent clade and were closely related to *C. locastrae* W.Y. Chuang et al. and *C. sandindaengensis* Mongkols et al. (Suppl. material 1: fig. S1; Fig. 1). *Hypocrella umbilicata* HKAS 126115, HKAS 154123, and HKAS 154124 formed a monophyletic clade within *Hypocrella* with high statistical support (BP = 93%, PP = 1.00) and were closely related to *H. calendulina* Hywel-Jones & Mongkols, *H. limushanensis* Hong Yu bis et al., *H. cf. discoidea*, and *Aschersonia napoleonae* Har. & Pat.

### Taxonomy

#### Ascopolyporus sinensis

Taxon classificationFungiSordariomycetesCordycipitaceae

Z.Q. Wang & Y.B. Wang sp. nov.

D84111A9-78D2-55CA-BE95-DA509EBD49E5

863421

Fig. 2

##### Etymology.

Named after the location, China, where this species was collected.

**Figure 2.**
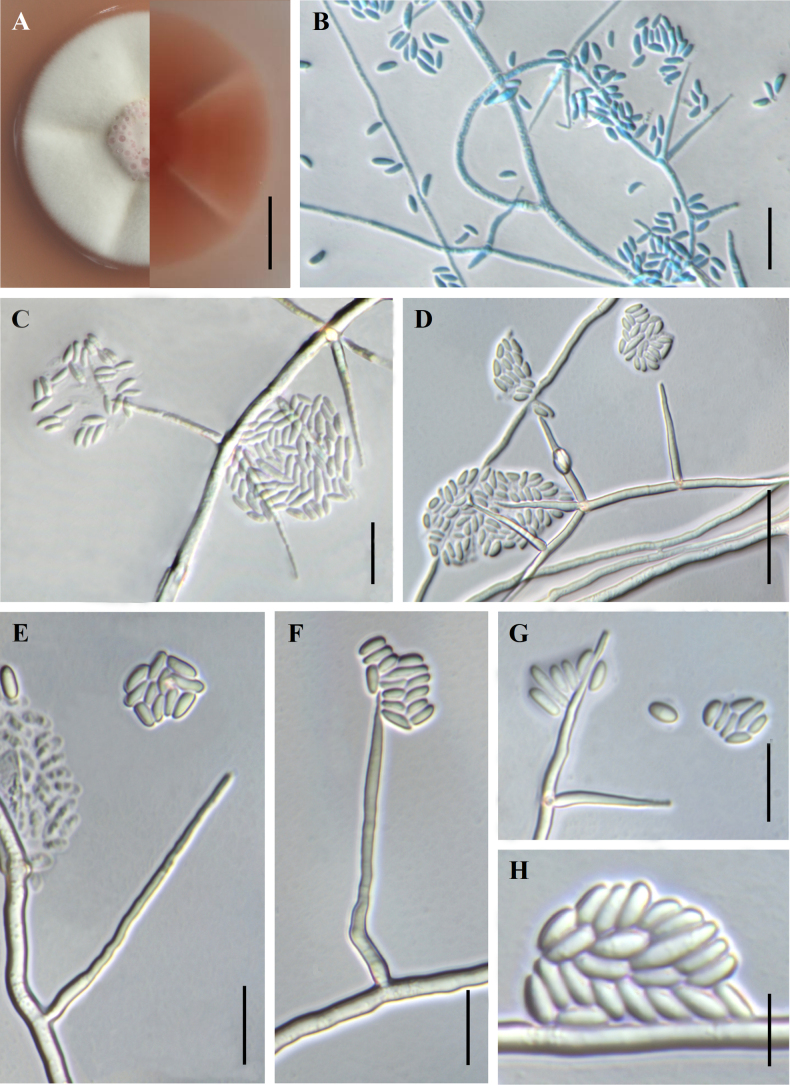
*Ascopolyporus sinensis*. **A**. Colony obverse and reverse on PDA at 25 °C after 14 days; **B–G**. Phialides and conidia; **H**. Conidia. Scale bars: 1 cm (**A**); 10 µm (**B–G**); 5 µm (**H**).

##### Type.

China • Yunnan Province, Pu’er City, Lianhuatang Village, 22°38'N, 100°58'E, alt. 1023 m, associated with *M. puerensis* Hong Yu bis et al. on the underside of living leaves of dicotyledonous plants. 12 Nov. 2025, Zhiqin Wang (HKAS 154757, holotype; KUNCC 11590, ex-holotype culture).

##### Description.

**Sexual morph**: Unknown. **Asexual morph**: Colonies on PDA attaining a diameter 35–37 mm after 14 days at 25 °C, regular wrinkles, white, cottony with high mycelial density, forming a circular bulge in the middle and surrounded by light pink water droplets; reverse pale pink, having pigment produced. Hyphae smooth‐walled, branched, septate, hyaline, 0.5–2.9 μm wide. Phialides verticillate, usually in whorls of two to three, or solitary on hyphae, 13.7–25.2 μm long; basal portion cylindrical, tapering gradually toward the apex, 1.1–2.5 μm wide at the base, 0.6–1.6 μm wide at the apex. Conidia smooth, hyaline, enteroblastic, fusiform to acerose, aggregated at the apex of phialides, 2.6–7.5 × 1.2–2.2 µm.

##### Habitat.

Associated with *M. puerensis* on the underside of living leaves of dicotyledonous plants.

##### Distribution.

China, Yunnan Province, Pu’er City.

##### Commentary.

Phylogenetically, *A. sinensis*KUNCC 11590 forms a separate clade at the base of the genus *Ascopolyporus* (Fig. 1). The most notable difference between *A. sinensis* and other species of *Ascopolyporus* is its habitat. All previously described species in the genus are associated with scale insects and are attached to the midribs of plant stems and leaves or directly parasitize plants (Thanakitpipattana et al. 2022; Yu et al. 2023). In contrast, *A. sinensis* was isolated from the stroma of *M. puerensis*. In addition, *A. sinensis* produces a pale purplish-pink pigment that diffuses into PDA medium, similar to *A. griseoperitheciatus* Khons., Thanakitp. & Luangsa-ard. Morphologically, however, the former can be distinguished from the latter by its shorter phialides (13.7–25.2 μm vs. 43–265 μm) (Thanakitpipattana et al. 2022). Among *Ascopolyporus* species, only *A. sinensis* and *A. tibetensis* F.M. Yu et al. have been recorded in China. These two species are phylogenetically distant, and because *A. tibetensis* has been reported only from sexual stromata in nature, with no successful strain isolation to date, morphological comparison between the two species is difficult.

#### Cordyceps anningensis

Taxon classificationFungiSordariomycetesCordycipitaceae

Z.Q. Wang & Y.B. Wang sp. nov.

767907A7-A34C-53DA-B87B-6914C38A28B6

863422

Fig. 3

##### Etymology.

Named after the location where this species was discovered.

**Figure 3.**
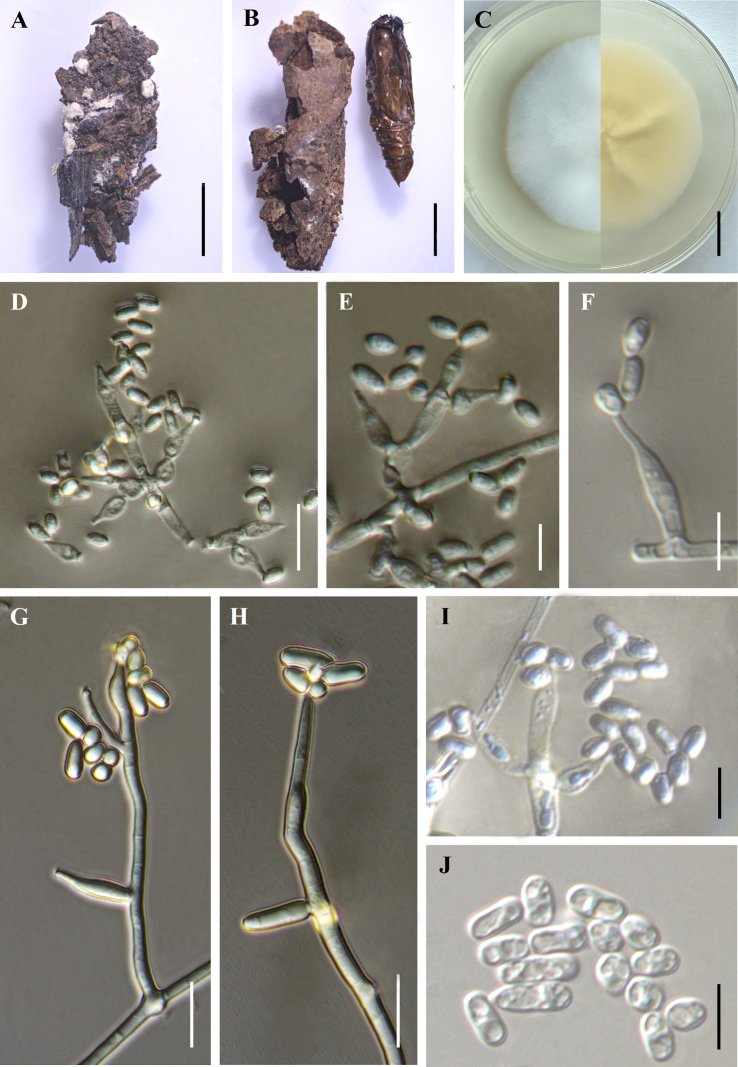
*Cordyceps anningensis*. **A, B**. Synnemata of the fungus arising from a larva of *Lepidoptera* inside a cocoon; **C**. Colonies, obverse and reverse, on PDA at 25 °C after 14 days; **D–I**. Phialides, solitary phialides on hyphae, and conidia; **J**. Conidia. Scale bars: 2 mm (**A, B**); 1 cm (**C**); 10 µm (**D, G, H**); 20 µm (**E, F, I, J**).

##### Type.

China • Yunnan Province, Anning City, Centipede Mountain, 24°53'N, 102°35'E, alt. 1930 m, found on a pupa of Lepidoptera inside a cocoon rolled in fallen leaves. 29 Jun. 2025, Zhiqin Wang (HKAS 154125, holotype; KUNCC 11591, ex-holotype culture).

##### Description.

**Sexual morph**: Unknown. **Asexual morph**: Several synnemata arising from the body of lepidopteran pupae, solitary, bearing a powdery, white mass of conidia at the apex. Colonies on PDA attaining a diameter of 44–46 mm after 14 days at 25 °C, white, cottony, slightly loose, with soft cottony aerial mycelium; reverse white to pale yellow. Hyphae smooth, septate, hyaline, 0.6–2.7 µm wide. Conidiophores smooth-walled, cylindrical, 3.4–9.5 × 1.5–2.9 µm. Phialides cylindrical to flask-shaped, solitary on hyphae, or whorled on hyphae and conidiophores, tapering gradually or abruptly toward the apex, 4.1–16.7 μm long, 0.9–2.7 μm wide at the base, 0.4–1.0 μm wide at the apex. Conidia often formed in an imbricate chain at the apex of phialides, hyaline, smooth-walled, elliptical to oblong, one-celled, 2.1–3.3 × 1.3–2.4 µm.

##### Habitat.

On pupae of *Lepidoptera* inside cocoons rolled in dry branches and fallen leaves.

##### Distribution.

China, Yunnan Province, Anning City.

##### Other material examined.

China • Yunnan Province, Anning City, Centipede Mountain. 24°53'N, 102°35'E, alt. 1930 m, found on a pupa of Lepidoptera inside a cocoon rolled in dry branches and fallen leaves. 29 Jun. 2025, Zhiqin Wang (HKAS 154126, paratype; KUNCC 11592, ex-paratype culture).

##### Commentary.

Phylogenetic analyses revealed that the two samples of *C. anningensis* grouped together, forming a distinct clade closely related to *C. locastrae* and *C. sandindaengensis* (Fig. 1). All three species are parasitic on lepidopteran pupae and occur only as asexual morphs in nature (Crous et al. 2023; Chuang et al. 2024). Morphologically, their conidia are often formed in imbricate chains at the apices of phialides. The host pupae of *C. anningensis* are inside cocoons rolled in dry branches and fallen leaves, whereas the hosts of the other two species are not wrapped in such materials. Furthermore, the synnemata of *C. anningensis* arise irregularly across the host body, whereas those of *C. sandindaengensis* emerge from the upper part of the host (Crous et al. 2023). *Cordyceps anningensis* produces smaller conidia (2.1–3.3 × 1.3–2.4 µm) than *C. locastrae* (3–3.5 × 2.5–3 µm) (Chuang et al. 2024).

#### Hypocrella umbilicata

Taxon classificationFungiSordariomycetesClavicipitaceae

Z.Q. Wang & Y.B. Wang sp. nov.

761C4DA0-2AC4-5AA2-8114-F97EBC93D46B

863423

Fig. 4

##### Etymology.

Named after the pulvinate stromata with a navel-like depression and notch at the center.

**Figure 4.**
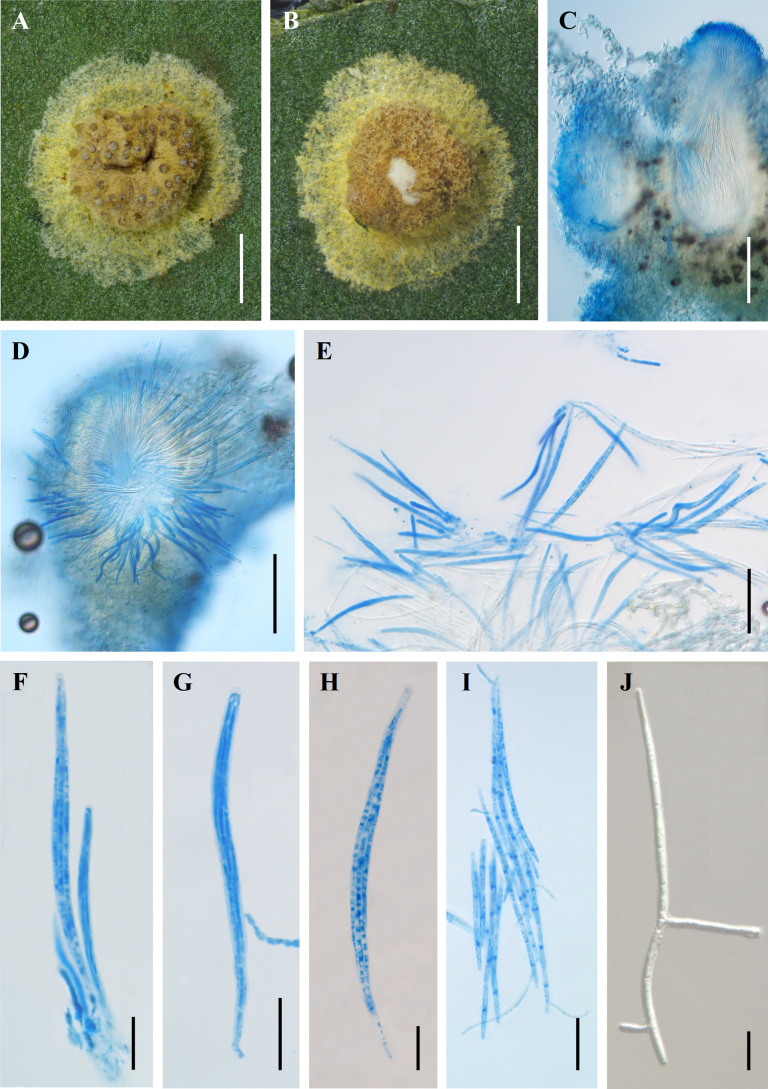
*Hypocrella umbilicata*. **A**. Stroma containing perithecia; **B**. Immature stroma; **C, D**. Perithecia; **E–H**. Asci; **I, J**. Germinating ascospores. Scale bars: 1 mm (**A, B**); 100 µm (**C, D**); 50 µm (**E**); 20 µm (**F–I**); 10 µm (**J**).

##### Type.

China • Yunnan Province, Pu’er City, Lianhuatang Village. 22°40'N, 100°32'E, alt. 984 m, on the underside of living leaves of dicotyledonous plants. 05 Aug. 2025, Zhiqin Wang (HKAS 154123, holotype).

##### Description.

**Sexual morph**: Stromata pulvinate, with a navel-like depression and notch at the center, 3.0–3.4 mm in diam, yellow to pale brown, surrounded by a yellow hypothallus, surface rough. Perithecia immersed in the stromata, scattered, absent from the central depressed area, with ostioles slightly projecting, dozens of perithecia per mature stroma, flask-shaped, 201–344 × 115–156 μm. Asci cylindrical, 66–200 × 2.9–9.3 μm, with an apical cap measuring 2.1–3.0 × 1.4–2.1 μm. Ascospores whole, non-fragmenting, hyaline, multiseptate, smooth-walled, filiform to long-fusiform, 51–123 × 1.4–3.4 μm. **Asexual morph**: Undetermined.

##### Habitat.

On whiteflies (*Aleyrodidae*, *Hemiptera*) occurring on the underside of living leaves of dicotyledonous plants.

##### Distribution.

China, Yunnan Province, Pu’er City.

##### Other material examined.

China • Yunnan, Pu’er City, Lianhuatang Village. 22°40'N, 100°32'E, alt. 984 m, on the underside of living leaves of dicotyledonous plants. 05 Aug. 2025, Zhiqin Wang (HKAS 126115, HKAS 154124, paratype).

##### Commentary.

Phylogenetic analyses resolved the three samples of *H. umbilicata* as a distinct, well-supported clade, showing a close phylogenetic relationship to *H. calendulina*, *H. limushanensis*, *H. cf. discoidea*, and *A. napoleonae* (Fig. 1). Because of the lack of comprehensive morphological documentation for *A. napoleonae* and *H. cf. discoidea*, direct morphological comparisons with these taxa are currently limited. Morphologically, *H. umbilicata* is easily distinguished from the closely related *H. calendulina* and *H. limushanensis* by its pulvinate stromata with a navel-like depression and notch at the center, whereas the stromata of the latter two species lack this feature (Mongkolsamrit et al. 2009; Wang et al. 2025). Furthermore, *H. calendulina* differs in having vividly marigold-orange stromata, whereas those of *H. umbilicata* are yellow to pale brown. *Hypocrella umbilicata* also has smaller perithecia (201–344 × 115–156 μm vs. 380–500 × 170–220 μm in *H. calendulina*) (Mongkolsamrit et al. 2009).

## Discussion

In the present study, the molecular data used for the phylogenetic analyses followed the frameworks established by Dong et al. (2022), Thanakitpipattana et al. (2022), and Crous et al. (2025). The phylogenetic analyses resolved the three newly proposed species as *A. sinensis*, *C. anningensis*, and *H. umbilicata*. *Cordyceps* has a remarkably broad host range, encompassing at least seven orders within *Arthropoda*, including *Araneae*, *Coleoptera*, *Dermaptera*, *Hemiptera*, *Hymenoptera*, *Lepidoptera*, and *Orthoptera* (Dong et al. 2022). Consistent with the topology generated by Dong et al. (2022), the phylogenetic inference recovered *Cordyceps* as comprising four statistically well-supported clades. Notably, *C. anningensis* clustered within a distinct subclade alongside *C. sandindaengensis* and *C. locastrae*, all of which share the common ecological trait of parasitizing lepidopteran pupae (Crous et al. 2023; Chuang et al. 2024). Yunnan Province is a hotspot for research on entomopathogenic fungi. Existing studies have shown that nearly 20 species of *Cordyceps* have been reported in this province (Dong 2023). Many species of the genus *Cordyceps* have considerable edible and medicinal value (Wang et al. 2020; Dong et al. 2022). Conducting a systematic investigation of the species resources of this group is of considerable theoretical and practical significance for subsequent resource development and utilization.

According to Index Fungorum (accessed 17 March 2026), approximately 120 names of *Hypocrella* s. l. and 92 names of *Aschersonia* s. l. have been recorded. However, many species have subsequently been reclassified into other genera. Chaverri et al. (2008) provided the most comprehensive review of *Hypocrella* s. l. since Petch (1921) and recognized 23 species within *Hypocrella* s. l. for which sexual–asexual connections to *Aschersonia* s. l. had been established. Nevertheless, the connections between many species within *Hypocrella* s. l. and the asexual *Aschersonia* s. l. remain unresolved because of the unavailability of corresponding molecular data. Members of *Hypocrella* s. l. are notable entomopathogens, frequently causing epizootics among populations of scale insects and whiteflies. These hosts typically colonize living leaves or, less commonly, branches of ferns, monocotyledonous plants, and dicotyledonous plants (Chaverri et al. 2005, 2008). Most species exhibit a primarily tropical distribution, whereas a few occur in subtropical regions (Petch 1921; Chaverri et al. 2005, 2008; Mongkolsamrit et al. 2009; Wang et al. 2025). *Hypocrella umbilicata* was found in a subtropical region, further contributing to the documented species diversity of this group in subtropical ecosystems.

*Ascopolyporus* species are generally regarded as epiphytic fungi associated with scale insects and plants, inhabiting substrates such as living or dead bamboo culms and the stems or leaf midribs of monocotyledonous and dicotyledonous plants (Thanakitpipattana et al. 2022; Yu et al. 2023). A notable affinity for bamboo is evident, with records of *A. albus* Mongkols et al., *A. gollmerianus* Henn., *A. polychrous*, *A. polyporoides* Möller, *A. puttemansii* Henn., *A. tibetensis* F.M. Yu et al., and *A. villosus* Möller reported on bamboo stems or branches (Yu et al. 2023). Currently, Index Fungorum (accessed 17 March 2026) lists 15 records for the genus. However, because of insufficient molecular and morphological data for several historically described species, only nine species were accepted by Thanakitpipattana et al. (2022), whereas 14 species were recognized in the broader assessment by Yu et al. (2023). The known geographical distribution of *Ascopolyporus* species spans Argentina, Bolivia, Brazil, China, Colombia, Costa Rica, Ecuador, Peru, and Thailand. Before this study, only *A. tibetensis* had been recorded from China. The newly introduced species *A. sinensis* thus represents the second confirmed occurrence of the genus in China and, notably, is the first species isolated from stromata of *M. puerensis*. This finding suggests that both the geographical distribution and host range of *Ascopolyporus* may be considerably broader than currently recognized.

## Supplementary Material

XML Treatment for Ascopolyporus sinensis

XML Treatment for Cordyceps anningensis

XML Treatment for Hypocrella umbilicata

## Additional information

### Artificial Intelligence (AI) use

The authors accept full responsibility for the content of the manuscript, including the disclosure of any use of AI.

No AI tools were used in the preparation of this manuscript.

### Author ORCIDs

Z.-Q. Wang https://orcid.org/0000-0001-9022-3635

Y.-X. Zhang https://orcid.org/0009-0006-8254-9225

Y.-B. Wang https://orcid.org/0000-0002-3305-9418

### Data availability

All of the data that support the findings of this study are available in the main text or Supplementary Information.

## Supplementary materials

Supplementary material 1 Phylogenetic tree comprising a comprehensive sampling of *Cordyceps* species Data type tif https://binary.pensoft.net/file/1723819This dataset is made available under the Open Database License (http://opendatacommons.org/licenses/odbl/1.0/). The Open Database License (ODbL) is a license agreement intended to allow users to freely share, modify, and use this Dataset while maintaining this same freedom for others, provided that the original source and author(s) are credited. Zhi-Qin Wang, Zi-Han Wang, Yu-Xin Zhang, Qian Song, Wen-Ya Shao, Wen-Li Li, Yuan-Bing Wang

Supplementary material 2 Suppl tables S1–S3 Data type pdf Explanation note **table S1**. List of species and corresponding GenBank accession numbers of sequences used in this study. **table S2**. PCR primers used in this study. **table S3**. The PCR amplification procedure for this study. References cited in tables S1, S2. https://binary.pensoft.net/file/1723820This dataset is made available under the Open Database License (http://opendatacommons.org/licenses/odbl/1.0/). The Open Database License (ODbL) is a license agreement intended to allow users to freely share, modify, and use this Dataset while maintaining this same freedom for others, provided that the original source and author(s) are credited. Zhi-Qin Wang, Zi-Han Wang, Yu-Xin Zhang, Qian Song, Wen-Ya Shao, Wen-Li Li, Yuan-Bing Wang
