# Supplementary figures and images for: Three new species in Cordycipitaceae and Clavicipitaceae (Hypocreales, Ascomycota) from Yunnan, China
Source: MycoKeys. 2026 Jul 23;137:257–72. doi: 10.3897/mycokeys.137.195207 (PMC13425056; doi:10.3897/mycokeys.137.195207)

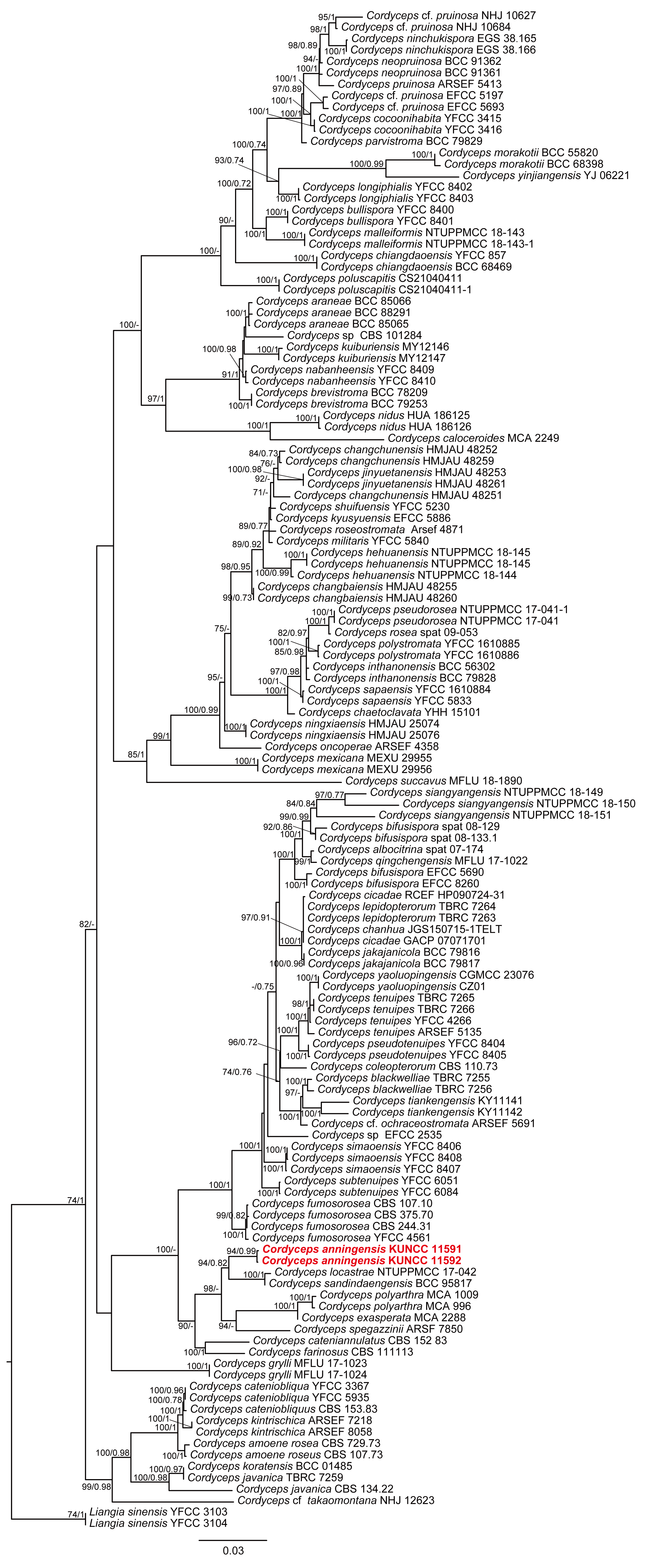

Supplement: Supplementary material 1 — Phylogenetic tree comprising a comprehensive sampling of Cordyceps species [file mycokeys-137-257-s001.tif]
