# Supplementary material for: Three new species in Cordycipitaceae and Clavicipitaceae (Hypocreales, Ascomycota) from Yunnan, China
Source: MycoKeys. 2026 Jul 23;137:257–72. doi: 10.3897/mycokeys.137.195207 (PMC13425056; doi:10.3897/mycokeys.137.195207)
Supplement: Supplementary material 2 — Suppl tables S1–S3 [file mycokeys-137-257-s002.pdf]

Supplementary material 2

Table S1. List of species and corresponding GenBank accession numbers of sequences used in this study.

Taxon	Voucher information	GenBank Accession Number					References
		nr*SSU*	nr*LSU*	*tef-1α*	*rpb1*	*rpb2*	
Aschersonia minutispora	BCC 17487	-	GU552151	GU552144	-	-	Mongkolsamrit et al. 2011
Aschersonia minutispora	BCC 20635	-	GU552149	GU552143	-	-	Mongkolsamrit et al. 2011
Aschersonia napoleonae	P.C. 737	-	AY986910	AY986936	DQ000337	-	Chaverri et al. 2005b
Ascopolyporus albus	BCC 48975	-	OL322048	OL322035	OL322056	OL322065	Thanakitpipattana et al. 2022
Ascopolyporus albus	BCC 48976	-	OL322049	OL322036	OL322057	OL322066	Thanakitpipattana et al. 2022
Ascopolyporus caulium	-	-	AF242354	-	-	-	Sullivan et al. 2000
Ascopolyporus galloides	BCC 25446	-	OL322042	OL322029	OL322053	OL322060	Thanakitpipattana et al. 2022
Ascopolyporus galloides	BCC 47981	-	OL322043	OL322030	OL322054	OL322061	Thanakitpipattana et al. 2022
Ascopolyporus galloides	BCC 48704	-	OL322044	OL322031	OL322055	OL322062	Thanakitpipattana et al. 2022
Ascopolyporus griseoperitheciatus	BCC 22358	-	OL322050	OL322037	-	OL322067	Thanakitpipattana et al. 2022
Ascopolyporus griseoperitheciatus	BCC 25788	-	OL322051	OL322038	OL322058	OL322068	Thanakitpipattana et al. 2022
Ascopolyporus khaoyaiensis	BCC 43314	-	OL322052	OL322039	-	OL322069	Thanakitpipattana et al. 2022
Ascopolyporus khaoyaiensis	BCC 43741	-	OL322041	OL322040	-	OL322070	Thanakitpipattana et al. 2022
Ascopolyporus philodendri	ARSEF 7354	-	AY886545	-	-	-	Bischoff et al. 2005
Ascopolyporus polychrous	P.C. 546	-	DQ118737	DQ118745	DQ127236	-	Chaverri et al. 2005a
Ascopolyporus purpuratus	BCC 88388	-	OL322046	OL322033	-	OL322064	Thanakitpipattana et al. 2022
Ascopolyporus purpuratus	BCC 88389	-	OL322047	OL322034	-	-	Thanakitpipattana et al. 2022
Ascopolyporus purpuratus	BCC 88430	-	OL322045	OL322032	OL322059	OL322063	Thanakitpipattana et al. 2022
Ascopolyporus sinensis	**KUNCC 11590**	-	**PZ240332**	**PZ244854**	-	**PZ252698**	**In this study**
Ascopolyporus tibetensis	HKAS 127119	-	OQ702345	OQ716551	-	-	Yu et al. 2023
Ascopolyporus tibetensis	HKAS 127120	-	OQ702344	OQ716550	-	-	Yu et al. 2023
Ascopolyporus tibetensis	HKAS 127121	-	OQ702346	OQ716552	-	-	Yu et al. 2023
Ascopolyporus villosus	ARSEF 6355	-	AY886544	DQ118750	DQ127241	-	Chaverri et al. 2005a
Blackwellomyces cardinalis	OSC 93609	AY184973	AY184962	DQ522325	DQ522370	DQ522422	Kepler et al. 2017
Blackwellomyces cardinalis	OSC 93610	AY184974	AY184963	EF469059	EF469088	EF469106	Kepler et al. 2017
Blackwellomyces pseudomilitaris	BCC 1919	MF416588	MF416534	MF416478	-	MF416440	Kepler et al. 2017
Blackwellomyces pseudomilitaris	BCC 2091	MF416589	MF416535	MF416479	-	MF416441	Kepler et al. 2017
Cordyceps anningensis	**KUNCC 11591**	**PZ240326**	**PZ240333**	**PZ244855**	**PZ244849**	**PZ252696**	**In this study**
Cordyceps anningensis	**KUNCC 11592**	**PZ240327**	**PZ240334**	**PZ244856**	**PZ244850**	**PZ252697**	**In this study**
Cordyceps amoene-rosea	CBS 107.73	AY526464	MF416550	MF416494	MF416651	MF416445	Luangsa-ard et al. 2005
Cordyceps amoene-rosea	CBS 729.73	MF416604	MF416551	MF416495	MF416652	MF416446	Luangsa-ard et al. 2005
Cordyceps blackwelliae	TBRC 7255	-	MF140703	MF140823	MF140772	MF140796	Mongkolsamrit et al. 2018
Cordyceps blackwelliae	TBRC 7256	-	MF140702	MF140822	MF140771	MF140795	Mongkolsamrit et al. 2018
Cordyceps brevistroma	BCC 78209	-	MT003044	MT017855	MT017817	MT017835	Mongkolsamrit et al. 2020
Cordyceps brevistroma	BCC 79253	-	MT003045	MT017856	-	MT017836	Mongkolsamrit et al. 2020
Cordyceps bullispora	YFCC 8400	OL468555	OL468575	OL473523	OL739569	OL473534	Dong et al. 2022
Cordyceps bullispora	YFCC 8401	OL468556	OL468576	OL473524	OL739570	OL473535	Dong et al. 2022
Cordyceps cateniannulata	CBS 152.83	AY526465	MG665226	JQ425687	-	-	Luangsa-ard et al. 2004; Mongkolsamrit et al. 2018
Cordyceps cateniobliqua	CBS 153.83	AY526466	-	JQ425688	-	MG665236	Luangsa-ard et al. 2004; Mongkolsamrit et al. 2018
Cordyceps cf. *ochraceostromata*	ARSEF 5691	EF468964	EF468819	EF468759	EF468867	EF468921	Kepler et al. 2012
Cordyceps cf. *takaomontana*	NHJ 12623	EF468984	EF468838	EF468778	EF468884	EF468932	Sung et al. 2007
Cordyceps chaetoclavata	YHH 15101	MN576722	MN576778	MN576948	MN576838	MN576894	Wang et al. 2020
Cordyceps changbaiensis	HMJAU 48255	-	MW893277	MZ616772	-	-	Hu et al. 2021
Cordyceps changbaiensis	HMJAU 48260	-	-	MZ616774	-	-	Hu et al. 2021
Cordyceps chiangdaoensis	BCC 68469	-	-	KT261403	-	-	Tasanathai et al. 2016; Mongkolsamrit et al. 2018
Cordyceps chiangdaoensis	YFCC 857	MW181781	MW173993	MW168234	MW168200	MW168217	Dong et al. 2022
Cordyceps coleopterorum	CBS 110.73	JF415965	JF415988	JF416028	JN049903	JF416006	Kepler et al. 2012
Cordyceps exasperata	MCA 2288	MF416592	MF416538	MF416482	MF416639	-	Kepler et al. 2017
Cordyceps farinosa	CBS 111113	AY526474	MF416554	MF416499	MF416656	MF416450	Luangsa-ard et al. 2004; Kepler et al. 2017
Cordyceps fumosorosea	YFCC 4561	MN576761	MN576817	MN576987	MN576877	MN576931	Wang et al. 2020
Cordyceps fumosorosea	CBS 244.31	MF416609	MF416557	MF416503	MF416660	MF416454	Kepler et al. 2017
Cordyceps hehuanensis	NTUPPMCC 18-144	-	MT974287	MW025861	MW025906	MW025943	Chuang et al. 2024
Cordyceps hehuanensis	NTUPPMCC 18-145	-	MT974289	MW025863	-	MW025945	Chuang et al. 2024
Cordyceps kintrischica	ARSEF 7218	-	-	GU734751	-	-	Humber et al. 2013
Cordyceps kintrischica	ARSEF 8058	-	-	GU734750	-	-	Humber et al. 2013

Taxon	Voucher information	GenBank Accession Number					References
		nr*SSU*	nr*LSU*	*tef-1α*	*rpb1*	*rpb2*	
Cordyceps koratensis	BCC 01485	GQ249957	GQ249981	GQ250031	-	-	Chuang et al. 2024
Cordyceps kuiburiensis	MY12146	-	MK968816	MK988032	MK988030	-	Crous et al. 2019
Cordyceps kuiburiensis	MY12147	-	MK968817	MK988033	MK988031	-	Crous et al. 2019
Cordyceps locastrae	NTUPPMCC 17-042	-	MT974256	MW025837	MW025883	MW025917	Chuang et al. 2024
Cordyceps malleiformis	NTUPPMCC 18-143	-	MT974282	MW025856	MW025902	MW025938	Chuang et al. 2024
Cordyceps malleiformis	NTUPPMCC 18-143-1	-	MT974283	MW025857	MW025903	MW025939	Chuang et al. 2024
Cordyceps mexicana	MEXU 29955	-	MN709062	MZ494672	MZ484384	-	Chuang et al. 2024
Cordyceps mexicana	MEXU 29956	-	-	MZ494673	-	-	Chuang et al. 2024
Cordyceps militaris	YFCC 5840	MN576763	MN576819	MN576989	MN576879	MN576933	Wang et al. 2020
Cordyceps ningxiaensis	HMJAU 25074	-	KF309671	-	-	-	Yan and Bau 2015
Cordyceps ningxiaensis	HMJAU 25076	-	KF309673	-	-	-	Yan and Bau 2015
Cordyceps pseudotenuipes	YFCC 8404	OL468559	OL468579	OL473527	OL739573	OL473538	Dong et al. 2022
Cordyceps pseudotenuipes	YFCC 8405	OL468560	OL468580	OL473528	OL739574	OL473539	Dong et al. 2022
Cordyceps poluscapitis	CS21040411	OM905727	OM905730	OM955655	OM955649	OM955652	Peng et al. 2023
Cordyceps poluscapitis	CS21040411-1	OM905729	OM905732	OM955657	OM955651	OM955654	Peng et al. 2023
Cordyceps polyarthra	MCA 996	MF416597	MF416543	MF416487	MF416644	-	Kepler et al. 2017
Cordyceps polyarthra	MCA 1009	MF416598	MF416544	MF416488	MF416645	-	Kepler et al. 2017
Cordyceps polystromata	YFCC 1610885	OQ878491	OQ878487	OQ868508	OQ868514	OQ868511	Wang et al. 2023
Cordyceps polystromata	YFCC 1610886	OQ878492	OQ878488	OQ868509	OQ868515	OQ868512	Wang et al. 2023
Cordyceps pruinosa	ARSEF 5413	AY184979	AY184968	DQ522351	DQ522397	DQ522451	Spatafora et al. 2007
Cordyceps sandindaengensis	BCC 95817	-	OQ540838	OQ473659	-	OQ473660	Crous et al. 2023
Cordyceps simaoensis	YFCC 8406	OL468561	OL468581	OL473529	OL739575	OL473540	Dong et al. 2022
Cordyceps simaoensis	YFCC 8407	OL468562	OL468582	OL473530	OL739576	OL473541	Dong et al. 2022
Cordyceps subtenuipes	YFCC 6051	MN576719	MN576775	MN576945	MN576835	MN576891	Wang et al. 2020
Cordyceps subtenuipes	YFCC 6084	MN576720	MN576776	MN576946	MN576836	MN576892	Wang et al. 2020
Cordyceps tenuipes	TBRC 7265	-	MF140707	MF140827	MF140776	MF140800	Mongkolsamrit et al. 2018
Cordyceps tenuipes	TBRC 7266	-	MF140708	MF140828	MF140777	MF140801	Mongkolsamrit et al. 2018
Cordyceps yaoluopingensis	CGMCC 23076	ON303837	ON311006	ON314456	ON314458	ON314454	Yang et al. 2022
Cordyceps yaoluopingensis	CZ01	ON303838	ON311007	ON314457	ON314459	ON314455	Yang et al. 2022
Corpulentispora magnispora	LC12469	-	-	MK336038	-	MK335986	Zhang et al. 2021
Corpulentispora magnispora	CGMCC 3.19304	-	-	MK336037	-	MK335985	Zhang et al. 2021
Corniculantispora dimorphum	CBS 363.86	AF339608	AF339559	EF468784	EF468890	-	Sung et al. (2007)
Corniculantispora dimorpha	CBS 345.37	KM283764	KM283788	KM283812	KM283834	KM283854	Park et al. (2016)
Conoideocrella fenshuilingensis	YHH CFFSL2310002	-	PP178583	PP776168	PP776158	-	Wang et al. 2025
Conoideocrella fenshuilingensis	YHH CFFSL2310003	-	PP178584	PP776169	PP776159	-	Wang et al. 2025
Conoideocrella tenuis	NHJ 6293	EU369112	EU369044	EU369029	EU369068	EU369087	Johnson et al. 2009
Conoideocrella tenuis	NHJ 6791	EU369113	EU369046	EU369028	EU369069	EU369089	Johnson et al. 2009
Hypocrella calendulina	BCC 20309	-	GU552154	KF016993	-	-	Mongkolsamrit et al. 2009
Hypocrella calendulina	BCC 20306	-	GU552148	KF016994	-	-	Mongkolsamrit et al. 2009
Hypocrella citrina	P.C. 606	-	EU392556	EU392640	EU392694	-	Chaverri et al. 2008
Hypocrella citrina	P.C. 598	-	EU392555	-	-	-	Chaverri et al. 2008
Hypocrella cf discoidea	I93-901D	-	EU392567	EU392646	EU392700	-	Chaverri et al. 2008
Hypocrella cf discoidea	I95-901D	-	EU392568	EU392647	EU392701	-	Chaverri et al. 2008
Hypocrella discoidea	BCC 2097	-	-	AY986945	DQ000346	-	Chaverri et al. 2008
Hypocrella discoidea	BCC 8237	-	DQ384937	DQ384977	DQ385000	DQ452461	Chaverri et al. 2008
Aschersonia luteola	BCC 9481	DQ372098	DQ384945	DQ384974	DQ384996	DQ452462	Crous et al. 2025
Aschersonia luteola	BCC 7865	DQ372099	DQ384946	DQ384975	DQ384997	-	Crous et al. 2025
Hypocrella disciformis	P.C. 655	-	EU392560	EU392643	EU392697	-	Chaverri et al. 2008
Hypocrella disciformis	P.C. 676	-	EU392566	EU392645	EU392699	-	Chaverri et al. 2008
Hypocrella hirsuta	P.C. 436.2	-	AY986922	AY986949	DQ000350	-	Chaverri et al. 2008
Hypocrella hirsuta	P.C. 543	-	EU392569	EU392648	EU392702	-	Chaverri et al. 2008
Hypocrella khonsanitii	BCC 71371	-	PQ560535	PQ585793	PQ585795	-	Crous et al. 2025
Hypocrella khonsanitii	BCC 69112	-	PQ560534	PQ585792	PQ585794	-	Crous et al. 2025
Hypocrella luteola	BCC 19360	-	GU552155	GU552147	-	-	Mongkolsamrit et al. 2009
Hypocrella limushanensis	YHH 2303015	-	OR828401	OR832089	OR837107	-	Wang et al. 2025
Hypocrella limushanensis	YHH 2303016	-	OR828402	OR832090	OR837108	-	Wang et al. 2025
Hypocrella siamensis	BCC 8105	DQ522537	DQ518752	DQ522317	DQ522363	DQ522411	Crous et al. 2025
Hypocrella umbilicata	**HKAS 126115**	**-**	**PZ240330**	**PZ244852**	**PZ244847**	**-**	**In this study**
Hypocrella umbilicata	**HKAS 154123**	**-**	**PZ240329**	**PZ244851**	**PZ244846**	**-**	**In this study**
Hypocrella umbilicata	**HKAS 154124**	**-**	**PZ240331**	**PZ244853**	**PZ244848**	**-**	**In this study**
Hypocrella viridans	P.C. 635	-	EU392572	EU392651	EU392705	-	Chaverri et al. 2008
Hypocrella viridans	P.C. 670	-	EU392574	EU392652	EU392706	-	Chaverri et al. 2008
Hypocrella yunnanense	YHH 2305020	-	OR828417	OR854260	OR837109	-	Wang et al. 2025
Hypocrella yunnanense	YHH 2305021	-	-	OR854261	OR837110	-	Wang et al. 2025

Taxon	Voucher information	GenBank Accession Number					References
		nr*SSU*	nr*LSU*	*tef-1α*	*rpb1*	*rpb2*	
Lecanicillium uredinophilum	KACC 47756	KM283759	KM283783	KM283807	KM283829	KM283849	Park et al. (2016)
Lecanicillium uredinophilum	KACC 44082	KM283758	KM283782	KM283806	KM283828	KM283848	Park et al. (2016)
Moelleriella alba	BCC 49409	-	JQ269646	KX254423	JQ256906	-	Mongkolsamrit et al. (2015)
Moelleriella alba	BCC 49492	-	JQ269645	KX254424	JQ256905	-	Mongkolsamrit et al. (2015)
Moelleriella chaiangmaiensis	BCC 18029	-	MT659360	MW091560	-	-	Khonsanit et al. 2021
Moelleriella chaiangmaiensis	BBH 33051	-	MT659362	MT672277	MT672269	-	Khonsanit et al. 2021
Moelleriella chaiangmaiensis	BCC 60941	-	MT659361	MT672278	MT672270	-	Khonsanit et al. 2021
Moelleriella jinuoana	YHH MJBP2309031	-	PP178643	PP776170	PP776160	-	Wang et al. 2025
Moelleriella jinuoana	YHH MJBP2309032	-	PP178644	PP776171	PP776161	-	Wang et al. 2025
Moelleriella jinuoana	YFCC MJBP23099451	-	PP178645	PP776172	PP776162	-	Wang et al. 2025
Orbiocrella petchii	NHJ 6240	EU369103	EU369038	EU369022	EU369060	EU369082	Johnson et al. 2009
Orbiocrella petchii	NHJ 6209	EU369104	EU369039	EU369023	EU369061	EU369081	Johnson et al. 2009
Paracorniculantispora sinensis	ZY06251	-	PV082870	PV171273	PV171145	PV171195	Chen et al. 2025
Paracorniculantispora sinensis	ZY06252	-	PV082871	PV171274	PV171146	PV171196	Chen et al. 2025
Pleurocordyceps aurantiaca	MFLUCC 17-2113	-	MG136910	MG136875	MG136866	MG136870	Xiao et al. 2023
Pleurocordyceps marginaliradians	MFLU 17-1582	-	-	MG136878	MG136869	MG271931	Xiao et al. 2023
Samuelsia geonomis	P.C. 614	-	EU392638	EU392692	EU392744	-	Chaverri et al. 2008
Samuelsia sheikhii	P.C. 686	-	EU392639	EU392693	EU392745	-	Chaverri et al. 2008
Samuelsia chalalensis	P.C. 560	-	EU392637	EU392691	EU392743	-	Chaverri et al. 2008
Samuelsia mundiveteris	BCC 40021	-	GU552152	GU552145	-	-	Mongkolsamrit et al. 2011
Samuelsia mundiveteris	BCC 40022	-	GU552153	GU552146	-	-	Mongkolsamrit et al. 2011
Samuelsia rufobrunnea	P.C. 613	-	AY986918	AY986944	DQ000345	-	Chaverri et al. 2008

Note: Bold accession numbers were generated for this study. The symbol "–" denotes no available data.

Table S2. PCR primers used in this study.

Gene	Primer	5'-Sequence-3'	Reference
nr*SSU*	NS1	GTAGTCATATGCTTGTCTC	White et al. 1990
	NS4	CTTCCGTCAATTCCTTTAAG	
nr*LSU*	LR5	ATCCTGAGGGAAACTTC	Vilgalys and Hester 1990; Rehner and Samuels 1994
	LR0R	GTACCCGCTGAACTTAAGC	
tef-1α	EF1α-EF	GCTCCYGGHCAYCGTGAYTTYAT	Bischoff et al. 2006; Sung et al. 2007
	EF1α-ER	ATGACACCRACRGCRACRGTYTG	
rpb1	RPB1-5'F	CAYCCWGGYTTYATCAAGAA	Bischoff et al. 2006; Sung et al. 2007
	RPB1-5'R	CCNGCDATNTCRTTRTCCATRTA	
rpb2	RPB2-5'F	CCCATRGCTTGTYYRCCCAT	Bischoff et al. 2006; Sung et al. 2007
	RPB2-5'R	GAYGAYMGWGATCAYTTYGG	

Table S3. The PCR amplification procedure for this study.

Step	nr*SSU*	nr*LSU*	*tef-1α*	*rpb1*	*rpb2*
1	95 °C, 4 min	95 °C, 4 min	95 °C, 4 min	95 °C, 4 min	95 °C, 4 min
2	94 °C, 1 min	94 °C, 50 s	94 °C, 50 s	94 °C, 50 s	94 °C, 50 s
3	52 °C, 1 min	60 °C, 50 s	52 °C, 50 s	53 °C, 50 s	55 °C, 50 s
4	72 °C, 90 s	72 °C, 55 s	72 °C, 1 min	72 °C, 1 min	72 °C, 90 s
5	GOTO step 2, 22×	GOTO step 2, 4×	GOTO step 2, 8×	GOTO step 2, 20×	GOTO step 2, 17×
6	94 °C, 1 min	94 °C, 50 s	94 °C, 50 s	94 °C, 50 s	94 °C, 50 s
7	51 °C, 1 min	56 °C, 50 s	51 °C, 50 s	52 °C, 50 s	51 °C, 50 s
8	72 °C, 90 s	72 °C, 55 s	72 °C, 1 min	72 °C, 1 min	72 °C, 90 s
9	GOTO step 6, 12×	GOTO step 6, 4×	GOTO step 6, 30×	GOTO step 6, 18×	GOTO step 6, 19×
10	72 °C, 10 min	94 °C, 50 s	72 °C, 10 min	72 °C, 10 min	72 °C, 10 min
11	12 °C, ∞	54 °C, 50 s	12 °C, ∞	12 °C, ∞	12 °C, ∞
12	–	72 °C, 55 s	–	–	–
13	–	GOTO step 10, 5×	–	–	–
14	–	94 °C, 50 s	–	–	–
15	–	52 °C, 50 s	–	–	–
16	–	72 °C, 55 s	–	–	–
17	–	GOTO step 14, 20×	–	–	–
18	–	72 °C, 8 min	–	–	–
19	–	12 °C, ∞	–	–	–

References

Bischoff JF, Chaverri P, White JF (2005) Clarification of the host substrate of *Ascopolyporus* and description of *Ascopolyporus philodendrus* sp. nov. Mycologia 97(3): 710–717. https://doi.org/10.1080/15572536.2006.11832800

Bischoff JF, Rehner SA, Humber RA (2006) *Metarhizium frigidum* sp. nov.: a cryptic species of *M. anisopliae* and a member of the *M. flavoviride* Complex. Mycologia 98(5): 737–745. https://doi.org/10.1080/15572536.2006.11832645

Chaverri P, Bischoff JF, Evans HC, Hodge KT (2005a) *Regiocrella*, a new entomopathogenic genus with a pycnidial anamorph and its phylogenetic placement in the *Clavicipitaceae*. Mycologia 97(6): 1225–1237. https://doi.org/10.1080/15572536.2006.11832732

Chaverri P, Bischoff JF, Liu M, Hodge KT (2005b) A new species of *Hypocrella*, *H. macrostroma*, and its phylogenetic relationships to other species with large stromata. Mycological Research 109(Pt 11): 1268–1275. https://doi.org/10.1017/s0953756205003904

Chaverri P, Liu M, Hodge KT (2008) A monograph of the entomopathogenic genera *Hypocrella*, *Moelleriella*, and *Samuelsia* gen. nov. (*Ascomycota*, *Hypocreales*, *Clavicipitaceae*), and their aschersonia-like anamorphs in the Neotropics. Studies in Mycology 60(1): 1–66. https://doi.org/10.3114/sim.2008.60.01

Chen WH, Shu HL, Li D, Liang JD, Sun CL, Zhao JH, Wijayawardene NN, Han YF, Tian WY (2025) Shedding light on the darkness: cryptic diversity of cordyceps-like fungi in karst regions of Guizhou Province, China. Mycosphere 16(1): 2887–2974. https://doi.org/10.5943/mycosphere/16/1/20

Chuang WY, Lin YC, Shrestha B, Luangsa-ard JJ, Stadler M, Tzean SS, Wu S, Ko CC, Hsieh SY, Wu ML, Wang SC, Shen TL, Ariyawansa HA (2024) Phylogenetic diversity and morphological characterization of cordycipitaceous species in Taiwan. Studies in Mycology 109(1): 1–56. https://doi.org/10.3114/sim.2024.109.01

Crous PW, Wingfield MJ, Lombard L, Roets F, Swart WJ, Alvarado P, Carnegie AJ, Moreno G, Luangsaard J, Thangavel R, Alexandrova AV, Baseia IG, Bellanger JM, Bessette AE, Bessette AR, De la Peña-Lastra S, García D, Gené J, Pham THG, Heykoop M, Malysheva E, Malysheva V, Martín MP, Morozova OV, Noisripoom W, Overton BE, Rea AE, Sewall BJ, Smith ME, Smyth CW, Tasanathai K, Visagie CM, Adamčík S, Alves A, Andrade JP, Aninat MJ, Araújo RVB, Bordallo JJ, Boufleur T, Baroncelli R, Barreto RW, Bolin J, Cabero J, Caboň M, Cafà G, Caffot MLH, Cai L, Carlavilla JR, Chávez R, de Castro RRL, Delgat L, Deschuyteneer D, Dios MM, Domínguez LS, Evans HC, Eyssartier G, Ferreira BW, Figueiredo CN, Liu F, Fournier J, Galli-Terasawa LV, Gil-Durán C, Glienke C, Gonçalves MFM, Gryta H, Guarro J, Himaman W, Hywel-Jones N, Iturrieta-González I, Ivanushkina NE, Jargeat P, Khalid AN, Khan J, Kiran M, Kiss L, Kochkina GA, Kolařík M, Kubátová A, Lodge DJ, Loizides M, Luque D, Manjón JL, Marbach PAS, Massola Jr NS, Mata M, Miller AN, Mongkolsamrit S, Moreau PA, Morte A, Mujic A, Navarro-Ródenas A, Németh MZ, Nóbrega TF, Nováková A, Olariaga I, Ozerskaya SM, Palma MA, Petters-Vandresen DAL, Piontelli E, Popov ES, Rodríguez A, Requejo Ó, Rodrigues ACM, Rong IH, Roux J, Seifert KA, Silva BDB, Sklenář F, Smith JA, Sousa JO, Souza HG, De Souza JT, Švec K, Tanchaud P, Tanney JB, Terasawa F, Thanakitpipattana D, Torres-Garcia D, Vaca I, Vaghefi N, van Iperen AL, Vasilenko OV, Verbeken A, Yilmaz N, Zamora JC, Zapata M, Jurjević Ž, Groenewald JZ (2019) Fungal planet description sheets: 951–1041. Persoonia 43: 223–425. https://doi.org/10.3767/persoonia.2019.43.06

Crous PW, Osieck ER, Shivas RG, Tan YP, Bishop-Hurley SL, Esteve-Raventós F, Larsson E, Luangsa-ard JJ, Pancorbo F, Balashov S, Baseia IG, Boekhout T, Chandranayaka S, Cowan DA, Cruz RHSF, Czachura P, De la Peña-Lastra S, Dovana F, Drury B, Fell J, Flakus A, Fotedar R, Jurjević Ž, Kolecka A, Mack J, Maggs-Kölling G, Mahadevakumar S, Mateos A, Mongkolsamrit S, Noisripoom W, Plaza M, Overy DP, Pitek M, Sandoval-Denis M, Vauras J, Wingfield MJ, Abell SE, Ahmadpour A, Akulov A, Alavi F, Alavi Z, Altés A, Alvarado P, Anand G, Ashtekar N, Assyov B, Banc-Prandi G, Barbosa KD, Barreto GG, Bellanger JM, Bezerra JL, Bhat DJ, Bilański P, Bose T, Bozok F, Chaves J, Costa-Rezende DH, Danteswari C, Darmostuk V, Delgado G, Denman S, Eichmeier A, Etayo J, Eyssartier G, Faulwetter S, Ganga KGG, Ghosta Y, Goh J, Góis JS, Gramaje D, Granit L, Groenewald M, Gulden G, Gusmão LFP, Hammerbacher A, Heidarian Z, Hywel-Jones N, Jankowiak R, Kaliyaperumal M, Kaygusuz O, Kezo K, Khonsanit A, Kumar S, Kuo CH, Laessøe T, Latha KPD, Loizides M, Luo SM, Maciá-Vicente JG, Manimohan P, Marbach PAS, Marinho P, Marney TS, Marques G, Martín MP, Miller AN, Mondello F, Moreno G, Mufeeda KT, Mun HY, Nau T, Nkomo T, Okrasińska A, Oliveira JPAF, Oliveira RL, Ortiz DA, Pawłowska J, Pérez-De-Gregorio MA, Podile AR, Portugal A, Privitera N, Rajeshkumar KC, Rauf I, Rian B, Rigueiro-Rodríguez A, Rivas-Torres GF, Rodriguez-Flakus P, Romero-Gordillo M, Saar I, Saba M, Santos CD, Sarma PVSRN, Siquier JL, Sleiman S, Spetik M, Sridhar KR, Stryjak-Bogacka M, Szczepańska K, Taşikn H, Tennakoon DS, Thanakitpipattana D, Trovão J, Türkekul A, van Iperen AL, van 't Hof P, Vasquez G, Visagie CM, Wingfield BD, Wong PTW, Yang WX, Yarar M, Yarden O, Yilmaz N, Zhang N, Zhu YN, Groenewald JZ (2023) Fungal planet description sheets: 1478–1549. Persoonia 50: 158–310. https://doi.org/10.3767/persoonia.2023.50.05

Crous PW, Catcheside DEA, Catcheside PS, Alfenas AC, Alfenas RF, Barreto RW, Lebel T, Balashov S, Broadbridge J, Jurjević Ž, De la Peña-Lastra S, Hoffmann R, Mateos A, Riebesehl J, Shivas RG, Soliz Santander FF, Tan YP, Altés A, Bandini D, Carriconde F, Cazabonne J, Czachura P, Gryta H, Eyssartier G, Larsson E, Pereira OL, Rigueiro-Rodríguez A, Wingfield MJ, Ahmad W, Bibi S, Denman S, Esteve-Raventós F, Hussain S, Illescas T, Luangsa-Ard JJ, Möller L, Mombert A, Noisripoom W, Olariaga I, Pancorbo F, Paz A, Piątek M, Polman-Short C, Suárez E, Afshan NS, Ali H, Arzanlou M, Ayer F, Barratt J, Bellanger JM, Bidaud A, Bishop-Hurley SL, Bohm M, Bose T, Campo E, Chau NB, Çolak ÖF, Cordeiro TRL, Cruz MO, Custódio FA, Couceiro A, Darmostuk V, Dearnaley JDW, de Azevedo Santiago ALCM, de Freitas LWS, Yáñez-Morales MJ, Domnauer C, Dentinger B, Dhileepan K, De Souza JT, Dovana F, Eberhardt U, Eisvand P, Erhard A, Fachada V, García-Martín A, Groenewald M, Hammerbacher A, Harms K, Haroon S, Haqnawaz M, Henriques

S, Hernández AJ, Jacobus LM, Jaen-Contreras D, Jangsantear P, Kaygusuz O, Knoppersen R, Kumar TKA, Lynch MJ, Mahiques R, Maraia GL, Marbach PAS, Mehrabi-Koushki M, Miller PR, Mongkolsamrit S, Moreau PA, Oberlies NH, Oliveira JA, Orlovich D, Pérez-Méndez AS, Pinto A, Raja HA, Ramírez GH, Raphael B, Rodrigues A, Rodrigues H, Ramos DO, Safi A, Sarwar S, Saar I, Sánchez RM, Santana JS, Scrace J, Sales LS, Silva LNP, Stryjak-Bogacka M, Tacconi A, Thanh VN, Thomas A, Thuy NT, Toome M, Valdez-Carrazco JM, van Vuuren NI, Vasey J, Vauras J, Vila-Viçosa C, Villarreal M, Visagie CM, Vizzini A, Whiteside EJ, Groenewald JZ (2025) Fungal planet description sheets: 1781–1866. Persoonia 54: 327–587. https://doi.org/10.3114/persoonia.2025.54.10

Dong QY, Wang Y, Wang ZQ, Tang DX, Zhao ZY, Wu HJ, Yu H (2022) Morphology and phylogeny reveal five novel species in the genus *Cordyceps* (*Cordycipitaceae*, *Hypocreales*) from Yunnan, China. Frontiers in Microbiology 13: 846909. https://doi.org/10.3389/fmicb.2022.846909

Hu JJ, Zhao GP, Tuo YL, Dai D, Guo DZ, Rao G, Qi ZX, Zhang ZH, Li Y, Zhang B (2021) Morphology and molecular study of three new Cordycipitoid fungi and its related species collected from Jilin Province, northeast China. MycoKeys 83: 161–180. https://doi.org/10.3897/mycokeys.83.72325

Humber RA, Rocha LFN, Inglis PW, Kipnis A, Luz C (2013) Morphology and molecular taxonomy of *Evlachovaea*-like fungi, and the status of this unusual conidial genus. Fungal Biology 117(1): 1–12. https://doi.org/10.1016/j.funbio.2012.10.001

Johnson D, Sung GH, Hywel-Jones NL, Luangsa-Ard JJ, Bischoff JF, Kepler RM, Spatafora JW (2009) Systematics and evolution of the genus *Torrubiella* (*Hypocreales*, *Ascomycota*). Mycological Research 113(Pt 3): 279–89. https://doi.org/10.1016/j.mycres.2008.09.008

Kepler RM, Sung GH, Ban S, Nakagiri A, Chen MJ, Huang B, Li Z, Spatafora JW (2012) New teleomorph combinations in the entomopathogenic genus *Metacordyceps*. Mycologia 104(1): 182–197. https://doi.org/10.3852/11-070

Kepler RM, Luangsa-Ard JJ, Hywel-Jones NL, Quandt CA, Sung GH, Rehner SA, Aime MC, Henkel TW, Sanjuan T, Zare R, Chen M, Li Z, Rossman AY, Spatafora JW, Shrestha B (2017) A phylogenetically-based nomenclature for *Cordycipitaceae* (*Hypocreales*). IMA Fungus 8(2): 335–353. https://doi.org/10.5598/imafungus.2017.08.02.08

Khonsanit A, Noisripoom W, Mongkolsamrit S, Phosrithong N, Luangsa-ard JJ (2021) Five new species of *Moelleriella* infecting scale insects (*Coccidae*) in Thailand. Mycological Progress 20: 847–867. https://doi.org/10.1007/s11557-021-01709-5

Luangsa-ard JJ, Hywel-Jones NL, Samson RA (2004) The polyphyletic nature of *Paecilomyces* sensu lato based on 18S-generated rDNA phylogeny. Mycologia 96(4): 773–780. https://doi.org/10.1080/15572536.2005.11832925

Luangsa-ard JJ, Hywel-Jones NL, Manoch L, Samson RA (2005) On the relationships of *Paecilomyces* sect. *Isarioidea* species. Mycological Research 109(Pt 5): 581–589. https://doi.org/10.1017/s0953756205002741

Mongkolsamrit S, Luangsa-Ard JJ, Spatafora JW, Sung GH, Hywel-Jones NL (2009) A combined ITS rDNA and beta-tubulin phylogeny of Thai species of *Hypocrella* with non-fragmenting ascospores. Mycological Research 113(Pt 6–7): 684–699. https://doi.org/10.1016/j.mycres.2009.02.004

Mongkolsamrit S, Luangsa-ard JJ, Hywel-Jones HJ (2011) *Samuelsia mundiveteris* sp. nov. from Thailand. Mycologia 103(4): 921–927. https://doi.org/10.3852/11-049

Mongkolsamrit S, Khonsanit A, Noisripoom W, Luangsa-ard JJ (2015) Two new entomogenous species of *Moelleriella* with perithecia in tubercles from Thailand. Mycoscience 56(1): 66–74. https://doi.org/10.1016/J.MYC.2014.03.002

Mongkolsamrit S, Noisripoom W, Thanakitpipattana D, Wutikhun T, Spatafora JW, Luangsa-ard JJ (2018) Disentangling cryptic species with isaria-like morphs in *Cordycipitaceae*. Mycologia 110(1): 230–257. https://doi.org/10.1080/00275514.2018.1446651

Mongkolsamrit S, Noisripoom W, Tasanathai K, Khonsanit A, Thanakitpipattana D, Himaman W, Kobmoo N, Luangsa-ard JJ (2020) Molecular phylogeny and morphology reveal cryptic species in *Blackwellomyces* and *Cordyceps* (*Cordycipitaceae*) from Thailand. Mycological Progress 19: 957–983. https://doi.org/10.1007/s11557-020-01615-2

Park MJ, Hong SB, Shin HD (2016) *Lecanicillium uredinophilum* sp. nov. associated with rust fungi from Korea. Mycotaxon 130(4): 997–1005. https://doi.org/10.5248/130.997

Peng XC, Xiao YP, Zhang Y, Chomnunti P, Tangtrakulwanich K, Wen TC (2023) *Cordyceps poluscapitis* sp. nov., an ant-pathogenic fungus from Guizhou, China. Phytotaxa 599(4): 239–251. https://doi.org/10.11646/phytotaxa.599.4.3

Rehner SA, Samuels GJ (1994) Taxonomy and phylogeny of *Gliocladium* analysed from nuclear large subunit ribosomal DNA sequences. Mycological Research 98(6): 625–634. https://doi.org/10.1016/S0953-7562(09)80409-7

Spatafora JW, Sung GH, Hywel-Jones NL, White JF (2007) Phylogenetic evidence for an animal pathogen origin of ergot and the grass endophytes. Molecular Ecology 16(8): 1701–1711. https://doi.org/10.1111/j.1365-294X.2007.03225.x

Sullivan RF, Bills GF, Hywel-Jones NL, White JF (2000) *Hyperdermium*: a new clavicipitalean genus for some tropical epibionts of dicotyledonous plants. Mycologia 92(5): 908–918. https://doi.org/10.2307/3761587

Sung GH, Hywel-Jones NL, Sung JM, Luangsa-ard JJ, Shrestha B, Spatafora JW (2007) Phylogenetic classification of *Cordyceps* and the clavicipitaceous fungi. Studies in Mycology 57: 5–59. https://doi.org/10.3114/sim.2007.57.01

Tasanathai K, Thanakitpipattana D, Noisripoom W, Khonsanit A, Kumsao J, Luangsa-ard JJ (2016) Two new *Cordyceps* species from a community forest in Thailand. Mycological Progress 15: 28. https://doi.org/10.1007/s11557-016-1170-3

Thanakitpipattana D, Mongkolsamrit S, Khonsanit A, Himaman W, Luangsa-ard JJ, Pornputtapong N (2022) Is *Hyperdermium* Congeneric with *Ascopolyporus*? Phylogenetic relationships of *Ascopolyporus* spp. (*Cordycipitaceae*, *Hypocreales*) and a new genus *Neohyperdermium* on scale insects in Thailand. Journal of Fungi 8(5): 516. https://doi.org/10.3390/jof8050516

Vilgalys R, Hester M (1990) Rapid genetic identification and mapping of enzymatically amplified ribosomal DNA from several *Cryptococcus* species. Journal of Bacteriology 172(8): 4238–4246. https://doi.org/10.1128/jb.172.8.4238-4246.1990

Wang Y, Dong QY, Luo R, Fan Q, Duan DE, Dao VM, Wang YB, Yu H (2023) Molecular phylogeny and morphology reveal cryptic species in the *Cordyceps militaris* complex from Vietnam. Journal of Fungi 9(6): 676. https://doi.org/10.3390/jof9060676

Wang YB, Wang Y, Fan Q, Duan DE, Zhang GD, Dai RQ, Dai YD, Zeng WB, Chen ZH, Li DD, Tang DX, Xu ZH, Sun T, Nguyen TT, Tran NL, Dao VM, Zhang CM, Huang LD, Liu YJ, Zhang XM, Yang DR, Sanjuan T, Liu XZ, Yang ZL, Yu H (2020) Multigene phylogeny of the family *Cordycipitaceae* (*Hypocreales*): new taxa and the new systematic position of the Chinese cordycipitoid fungus *Paecilomyces hepiali*. Fungal Diversity 103: 1–46. https://doi.org/10.1007/s13225-020-00457-3

Wang ZQ, Yang ZL, Zhao J, Ma JM, Tang DX, Liang ZL, Li JH, Zhou XM, Yu H (2025) Taxonomy and phylogeny of entomopathogenic fungi from China—revealing two new genera and thirteen new species within *Clavicipitaceae* (*Hypocreales*, *Ascomycota*). MycoKeys 117: 121–169. https://doi.org/10.3897/mycokeys.117.140577

White TJ, Bruns TD, Lee SB, Taylor JW (1990) Amplification and direct sequencing of fungal ribosomal RNA genes for phylogenetics. In: Innis MA, Gelfand DH, Sninsky JJ, White TJ (Eds) PCR protocols: a guide to methods and applications. Academic, New York, 315–322. https://doi.org/10.1016/B978-0-12-372180-8.50042-1

Xiao YP, Wang YB, Hyde KD, Eleni G, Sun JZ, Yang Y, Meng J, Yu H, Wen TC (2023) *Polycephalomycetaceae*, a new family of clavicipitoid fungi segregates from *Ophiocordycipitaceae*. Fungal Diversity 120: 1–76. https://doi.org/10.1007/s13225-023-00517-4

Yan JQ, Bau T (2015) *Cordyceps ningxiaensis* sp. nov., a new species from dipteran pupae in Ningxia Hui autonomous region of China. Nova Hedwigia 100(1–2): 251–258. https://doi.org/10.1127/nova_hedwigia/2014/0222

Yang Y, Xiao YP, Cheng YN, Xu FY, Han HM, Luo X (2022) *Cordyceps yaoluopingensis* sp. nov., a new entomopathogenic species from China. Phytotaxa 571(3): 278–290. https://doi.org/10.11646/phytotaxa.571.3.2

Yu FM, Wei DP, Zhao Q, Tang SM, Luangharn T (2023) *Ascopolyporus tibetensis* (*Cordycipitaceae*, *Hypocreales*): a new species from Tibet, China. Phytotaxa 592(2): 088–098. https://doi.org/10.11646/phytotaxa.592.2.2

Zhang ZF, Zhou SY, Eurwilaichitr L, Ingsriswang S, Raza M, Chen Q, Zhao P, Liu F, Cai L (2021) Culturable mycobiota from Karst caves in China II, with descriptions of 33 new species. Fungal Diversity 106: 29–136. https://doi.org/10.1007/s13225-020-00453-7
